# Solution NMR assignment of the C-terminal domain of human chTOG

**DOI:** 10.1007/s12104-018-9812-9

**Published:** 2018-03-26

**Authors:** Elena Rostkova, Selena G. Burgess, Richard Bayliss, Mark Pfuhl

**Affiliations:** 1 Cardiovascular and Randall Division, King’s College London, Guy’s Campus, London, SE1 1UL UK; 2 Faculty of Biological Sciences, School of Molecular and Cellular Biology, Astbury Centre for Structural Molecular Biology, University of Leeds, Leeds, LS2 9JT UK

**Keywords:** Mitosis, Kinetochore, TACC3, ChTOG, Cell cycle

## Abstract

The microtubule regulatory protein colonic and hepatic tumor overexpressed gene (chTOG), also known as cytoskeleleton associated protein 5 (CKAP5) plays an important role in organizing the cytoskeleton and in particular in the assembly of k-fibres in mitosis. Recently, we dissected the hitherto poorly understood C-terminus of this protein by discovering two new domains—a cryptic TOG domain (TOG6) and a smaller, helical domain at the very C-terminus. It was shown that the C-terminal domain is important for the interaction with the TACC domain in TACC3 during the assembly of k-fibres in a ternary complex that also includes clathrin. Here we now present the solution NMR assignment of the chTOG C-terminal domain which confirms our earlier prediction that it is mainly made of α-helices. However, the appearance of the ^1^H–^15^N HSQC spectrum is indicative of the presence of a considerable amount of unstructured and possibly flexible portions of protein in the domain.

## Biological context

The human protein colonic and hepatic tumor overexpressed gene (chTOG), also known as CKAP5, and its *Drosophila melanogaster* homologue, MSPS (mini spindles) are members of the XMAP215 protein family (Ohkura et al. 2001). These proteins vary considerably in size and consist of an N-terminal region comprising 2, 3 or 5 highly conserved TOG domains followed by a C-terminal region that is much more diverse in sequence and varying in length. For most members of the XMAP215 protein family this C-terminal region is poorly described and studied. Furthermore, while the organisation of the N-terminal TOG domain array is highly conserved between yeast and higher eukaryotes, this is not the case for the C-terminus (Gard et al. 2004). e.g., the yeast homologue, Stu2p contains a C-terminal coiled coil dimerisation domain while family members in higher eukaryotes are monomeric (van Breugel et al. 2003). Yet it is through this part of the protein that a large number of interactions to other regulatory proteins occurs, defining the specific and distinct function of each family member. Members of this family function as tubulin polymerases and are thus important for remodelling of the microtubule cytoskeleton (Al-Bassam and Chang 2011). They target microtubule plus ends and mutants in this protein family cause reduced microtubule growth rates and aberrant spindle morphologies (Currie et al. 2011). In mitosis, they are found in at least three distinct pools: at centrosomes, associated with microtubule plus ends and in kinetochore fibres (Gutiérrez-Caballero et al. 2015). Indeed, XMAP215 proteins play an important role in the assembly of kinetochore fibres as they help to crosslink adjacent microtubules in a complex with clathrin and transforming acidic coiled-coil protein 3 (TACC3). Assembly of the complex is regulated by Aurora-A phosphorylation of TACC3 as phosphorylation on Ser558 (TACC3 numbering) is required for the subsequent interaction between clathrin and TACC3 (Hood et al. 2013). The specific distribution of all three proteins during mitosis differs slightly with chTOG more prominent at centrosomes whereas TACC3 is more evident on spindle microtubules. Both clathrin and TACC3 are required for the correct localisation of the complex yet neither can independently bind to microtubules in vitro. To improve our understanding of the events regulating complex assembly, there has been considerable work to better define the interaction sites of the three proteins. It has been shown that a short stretch of polypeptide chain near the TACC domain in TACC3 and the ankle region of clathrin combine to create a composite binding site for microtubules, which is coordinated by phosphorylation of TACC3 by Aurora-A (Hood et al. 2013). chTOG is recruited to this complex via its interaction with TACC3. Previous work has shown that the C-terminus of chTOG (residues 1517–1957) is sufficient for binding to TACC3 (Hood et al. 2013; Thakur et al. 2014). We initially characterised the equivalent region in MSPS (residues 1591–1941) by NMR spectroscopy. The NMR data showed that this fragment contains two distinct domains (Hood et al. 2013). Using a combination of more detailed NMR analysis and sequence similarity searches the first and larger of these was identified as a sixth, cryptic, TOG domain (TOG6; residues 1591–1850 in MSPS, residues 1517–1802 in chTOG) (Hood et al. 2013; Burgess et al. 2015). The remaining, shorter domain at the very C-terminus of both proteins (residues 1860–1941 in MSPS, 1817–1957 in chTOG) did not show any similarity to a known fold. The preliminary NMR analysis of this region in MSPS suggested the presence of 4 α-helices so that this fragment was termed provisionally the ‘4 helix domain’.

Efforts to study this domain from MSPS on its own failed because of its tendency to degrade already in the *E. coli* cells during recombinant expression. Moreover, there were similar problems of degradation with the TACC domain of the drosophila homologue of TACC3, dTACC. As the chTOG/MSPS CTD is implicated in interactions with the TACC domain it was therefore decided to characterize the CTD of chTOG instead. The importance of the very C-terminal residues in chTOG for binding to TACC3 was demonstrated by the loss of interaction upon deletion of residues 1932–1957 (Gutiérrez-Caballero et al. 2015). These results suggest that the binding site for the TACC domain resides in the CTD and not in the cryptic TOG6 domain. This makes a study of the chTOG CTD an important step in dissecting k-fibre assembly.

## Methods and experiments

Residues 1817–1957 of chTOG were cloned into pETM6T1 for expression as a NusA fusion protein (Harrison 2000) with a N-terminal 6-His-tag and a TEV site between NusA and the chTOG fragment. Protein was expressed in *E. coli* BL21 cells over night at a temperature of 18 °C. NMR samples were prepared in a buffer of 20 mM HEPES, 150 mM Glutamic acid/Arginine, 2 mM DTT pH 7.2 with protein concentrations between 200 and 500 µM. Backbone assignment of the CTD was performed using HNCA, HNCACB, HN(CO)CACB, HN(CO)CA, H(CCCO)NH, (H)C(CCO)NH and HNCO experiments recorded at 700, 800 and 950 MHz on Bruker Avance spectrometers at 25 °C. Side chain resonance assignment was initialized using the backbone-sidechain TOCSY experiments H(CCCO)NH and (H)C(CCO)NH and completed using a combination of HCCH TOCSY and ^13^C NOESY-HSQC experiments. Spectra were processed with Topspin 3.1 (Bruker) and all assignments were performed with CCPN analysis 2.4 (Vranken et al. 2005).

## Assignments and data deposition

**Fig. 1:**
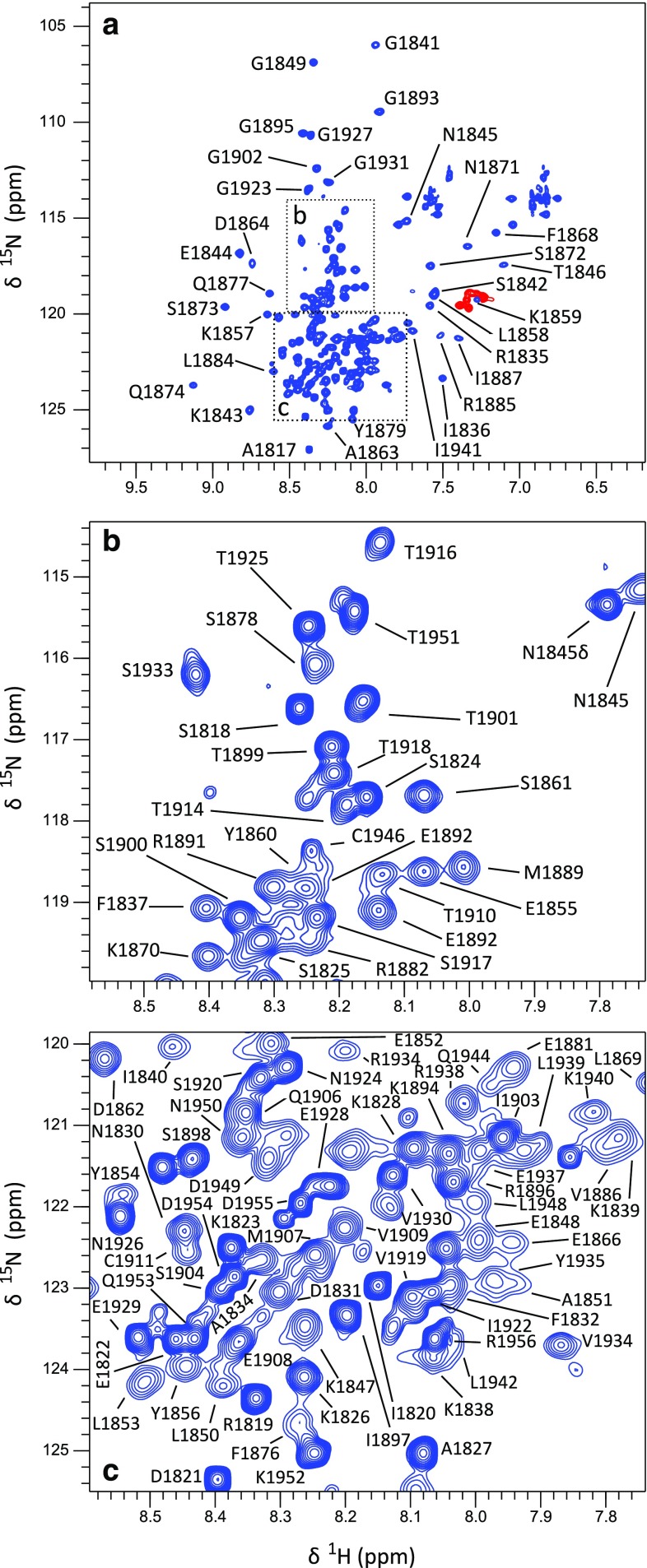
^1^H–^15^N TROSY spectrum of 400 µM chTOG 1817–1957 comprising the C-terminal domain recorded at a temperature of 298 K and a field of 800 MHz. Well resolved peaks are labelled with residue type and number in part (**a**) while peaks in the crowded central region are labelled in sub spectra (**b, c**). Positions of sub spectra (**b, c**) in the overall spectrum (**a**) are indicated by dashed boxes. Red peaks indicate peaks with negative intensity caused by aliasing of the ^15^N resonance frequency. They are the resonances of the arginine Hε/Nε groups

**Fig. 2:**
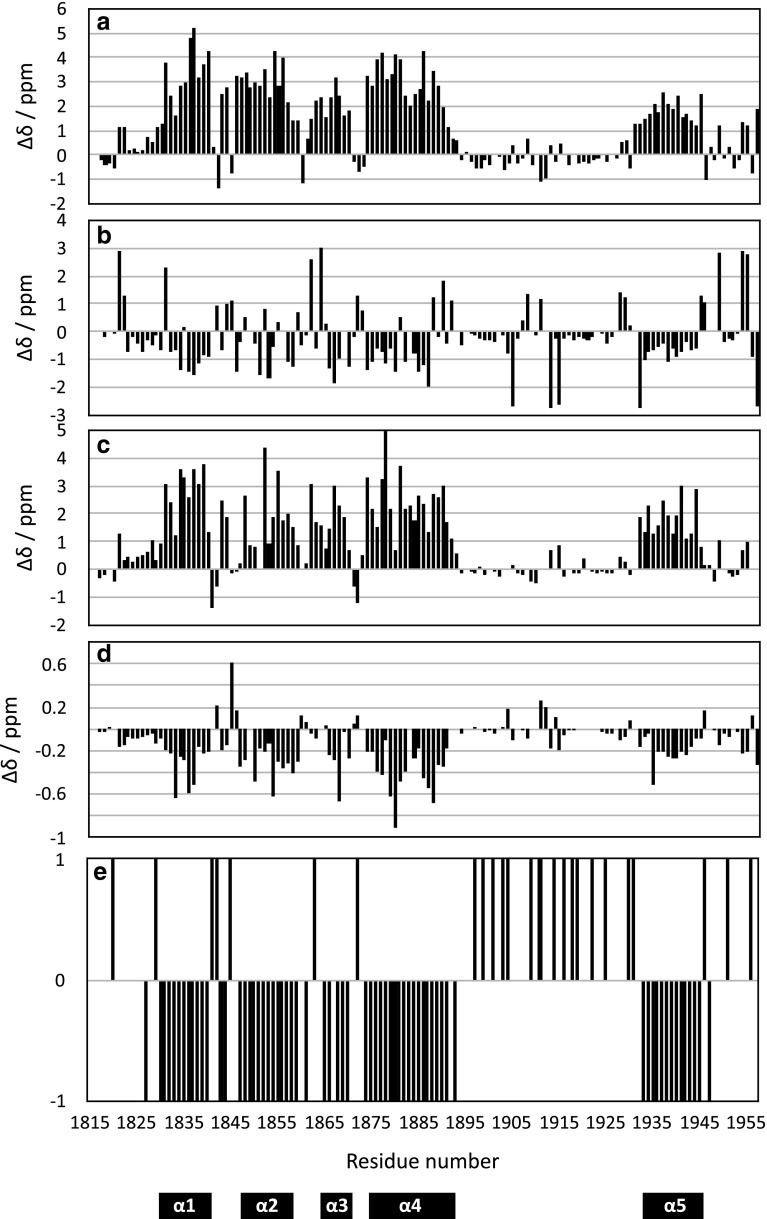
Secondary chemical shifts and chemical shift index (CSI) (Wishart and Sykes 1994). All values were calculated using CCPN analysis version 2.4 and figures were generated in apple numbers. **a** Cα, **b** Cβ, **c** C′, **d** Hα, **e** CSI. Positions of secondary structure elements based on the chemical shift analysis are indicated as bars

The chTOG CTD expressed well in standard *E. coli* BL21 cells with good yields of about 10 mg in 1 L of LB. The domain gives good spectra (see Fig. 1) even though there appears to be a substantial variation in peak intensity and distribution: numerous peaks of very high intensity cluster around the random coil region while a slightly larger number are more widely distributed which suggests a considerable amount of at least partially disordered protein in the domain. This is confirmed by the analysis of ^13^C and ^1^H secondary chemical shifts where we can clearly confirm the existence of the predicted four helices from our previous work on the MSPS homologue of the domain (Fig. 2). In addition, there is evidence for a fifth helix at the C-terminus even though the secondary chemical shifts are slightly weaker than those for the other four helices. In between we see very weak secondary chemical shifts suggestive of a largely disordered, flexible protein in good agreement with the appearance of the ^1^H–^15^N HSQC experiment. Finally, the presence of at least 6 sidechain arginine He/Ne peaks (shown in red in Fig. 1a)—out of a total of nine possible ones—should be noted given the relatively high pH value of 7.2. These suggest the presence of a quite high proportion of arginines in protective interactions such as salt bridges or hydrogen bonds.

The CTD domain construct comprises 141 amino acids (residues 1817–1957). It was possible to find assignments for all of these even though backbone amide peaks were missing for three of them (F1875, V1912, R1945). Out of a total of 141 backbone nitrogens, 141 α carbons, 131 β carbons, 126 γ carbons, 86 δ carbons, 26 ε carbons and 141 backbone carbonyls a total of 132 (94%), 141 (100%), 131 (100%), 102 (81%), 65 (76%), 25 (96%) and 132 (94%), respectively, could be assigned. 405 out of a total of 423 backbone resonances (96%) and 543 out of a total of 647 sidechain proton resonances (84%) were assigned. In terms of sidechain nitrogen containing groups, all six asparagine δ NH_2_ groups, three of five glutamine ε NH_2_ groups and two out of nine arginine ε groups could be assigned. The assignment has been deposited with the BMRB, accession code 27235.
